# The prevalence and survival of pulmonary hypertension due to left heart failure: A retrospective analysis of a multicenter prospective cohort study

**DOI:** 10.3389/fcvm.2022.908215

**Published:** 2022-08-02

**Authors:** Yangyi Lin, Lingpin Pang, Shian Huang, Jieyan Shen, Weifeng Wu, Fangming Tang, Weiqing Su, Xiulong Zhu, Jingzhi Sun, Ruilin Quan, Tao Yang, Huijun Han, Jianguo He

**Affiliations:** ^1^Department of Pulmonary Vascular Disease, State Key Laboratory of Cardiovascular Disease, Fuwai Hospital, National Center for Cardiovascular Diseases, Chinese Academy of Medical Sciences and Peking Union Medical College, Beijing, China; ^2^Cardiovascular Medicine Center, Affiliated Hospital of Guangdong Medical University, Zhanjiang, China; ^3^Department of Cardiology, Renji Hospital, Shanghai Jiao Tong University School of Medicine, Shanghai, China; ^4^Department of Cardiology, The First Affiliated Hospital of Guangxi Medical University, Nanning, China; ^5^Department of Cardiology, Nongken Central Hospital of Guangdong Province, Zhanjiang, China; ^6^Department of Cardiology, Lianjiang People’s Hospital, Lianjiang, China; ^7^Department of Cardiology, People’s Hospital of Gaozhou, Gaozhou, China; ^8^Department of Cardiology, Affiliated Hospital of Jining Medical University, Jining, China; ^9^Department of Epidemiology and Biostatistics, Institute of Basic Medical Sciences, Chinese Academy of Medical Sciences, School of Basic Medicine, Peking Union Medical College, Beijing, China

**Keywords:** pulmonary hypertension, left heart failure, coronary artery disease, prevalence, mortality

## Abstract

**Background:**

Pulmonary hypertension due to left heart failure (PH-LHF) is currently the most common form of pulmonary hypertension (PH) encountered in clinical practice. Despite significant advances that have improved our understanding of PH-LHF over the past two decades, the mortality is still high in recent decades. This study aimed to describe the prevalence and survival of patients with PH-LHF, and explored the potential risk factors which may predict the prognosis of PH-LHF.

**Methods:**

A retrospective analysis of a prospective cohort study of left heart failure (LHF) patients who underwent right heart catheterization (RHC) between January 2013 and November 2016 was performed. The endpoint was all-cause mortality. Follow-ups were performed every 6 months ± 2 weeks.

**Results:**

A total of 480 patients with LHF were enrolled, with 215 (44.8%) having PH-LHF. The proportion of PH-LHF was significantly lower in coronary artery disease (CAD) group than without CAD (41.3 vs. 57.8%, *p* = 0.003). However, multivariable logistic regression analysis revealed that CAD was not associated with PH-LHF (Adjusted OR: 1.055, 95% CI: 0.576 – 1.935, *p* = 0.862). 75 of 215 (34.9%) patients with PH-LHF died during a median follow-up period of 84.6 months. The 1-, 3-, 5-, and 8-year survival rates of all PH-LHF patients were 94.3, 76.9, 65.8, and 60.2%, respectively. New York Heart Association Functional Class (NYHA FC), hemoglobin, and systolic pulmonary artery pressure (sPAP) were associated with mortality of PH-LHF in multivariate Cox analysis.

**Conclusion:**

PH is commonly identified in patients with LHF, with a prevalence of approximately 45%. The mortality is still high in patients with PH-LHF. NYHA FC, hemoglobin, and sPAP are independent risk predictors of mortality for PH-LHF. These findings may be useful for risk stratification in future clinical trial enrollment.

## Introduction

Pulmonary hypertension (PH) has become an increasingly common global health issue. It is estimated having the prevalence of about 1% of the global population increases up to 10% in individuals older than 65 years (1). The clinical classification of PH is categorized into five groups, PH due to left heart failure (PH-LHF) is categorized as group 2, and is defined as post-capillary PH [mean pulmonary arterial pressure (mPAP) ≥ 25 mmHg and pulmonary arterial wedge pressure (PAWP) > 15 mmHg] (2). The overall incidence of left heart failure (LHF) is increasing due to the rapid global rise in the number of people older than 65 years, LHF is becoming a leading cause of PH, affecting around 5% of individuals aged 65 years or older (1, 3, 4).

Pulmonary hypertension due to left heart failure is a frequent co-morbidity of left ventricular diastolic dysfunction heart failure with preserved ejection fraction (HFpEF) or heart failure with reduced ejection fraction (HFrEF), the prevalence reported in previous studies ranges from 40 to 75% for PH-HFrEF and from 36 to 83% for PH-HFpEF (1, 5). However, this prevalence is derived from a non-uniform application of the gold diagnostic standard (RHC). For example, some reports depend on pulmonary artery systolic pressure (PASP) measured by echocardiography or use different diagnostic criteria (6, 7). Therefore, the true prevalence of PH-LHF is unclear.

The mortality of patients with LHF has significantly decreased over the past two decades (8). However, the mortality of PH-LHF is still high in recent years (3, 9). PH-LHF has a poorer survival when compared with patients with pulmonary arterial hypertension (PAH) (3). PAH has several practical risk tables for stratifying prognosis that provides treatment goals and follow-up strategy (2, 10). However, the practical and reliable risk table for PH-LHF is unavailable. Identifying potential risk factors may be helpful to alleviate the problem of high mortality in PH-LHF patients. Accordingly, the aims of this study were: (1) to describe the prevalence of PH in patients with LHF; (2) to plan and conduct a long-term follow-up of patients with PH-LHF to estimate survival; and (3) to explore the potential risk factors which may predict the death of PH-LHF.

## Materials and methods

### Study design and participants

**FIGURE 1:**
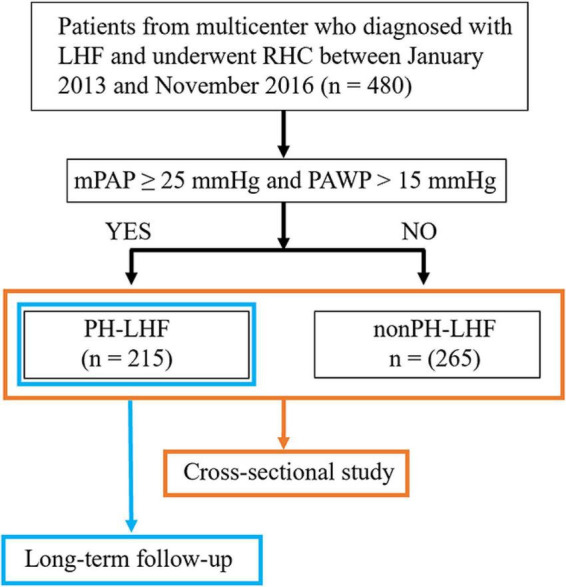
The study design and flowchart for the selection of patients. LHF, left heart failure; RHC, right heart catheterization; mPAP, mean pulmonary artery pressure; PAWP, pulmonary artery wedge pressure; PH-LHF, pulmonary hypertension due to left heart failure.

We conducted a retrospective analysis of a prospective, multicenter registry study of LHF patients who underwent right heart catheterization (RHC) between January 2013 and November 2016. The study design and the flowchart of patient selection are displayed in Figure 1. The study protocol was approved by the Institutional Review Board of Fuwai Hospital (Approval No. 2012-401), conducted as per the Declaration of Helsinki, and was registered on ClinicalTrials.gov (Identifier: NCT02164526). Written informed consent was obtained from all enrolled patients.

Patients were enrolled in the study according to the following criteria: (1) confirmed diagnosis with LHF according to the guideline on heart failure at that time (11). (2) Patients who underwent RHC between January 2013 and November 2016. Patients with any of the following criteria were excluded: (1) hypertrophic obstructive cardiomyopathy; (2) right ventricular outflow tract stenosis; (3) pericardial disease; (4) patients with chronic lung disease; and (5) HF due to valvular heart disease.

### Measurements and data collection

Echocardiography, electrocardiography, pulmonary function tests, ventilation/perfusion scintigraphy lung scan, chest X-ray, high-resolution CT of the chest, pulmonary angiography (if necessary), RHC, medical history, clinical symptoms, signs, and laboratory results were assessed to rule out PH in other groups. Biochemical blood works were performed within 24 h of admission. The blood pressure, heart rate, echocardiography, and biochemical parameters were obtained from the first measurement on admission. RHC was conducted to confirm a physician’s diagnosis of suspected PH-LHF with an elevated systolic pulmonary arterial systolic pressure measured by echocardiography or to assess and monitoring of hemodynamics, or conducted in patients whose exercise capacity decreased despite optimal guideline-directed treatment. RHC and left heart catheterization were used to obtain hemodynamic parameters, while quantitative coronary angiography was used to measure angiographic parameters.

Pulmonary hypertension is an increase in mean pulmonary arterial pressure (mPAP) ≥ 25 mmHg at rest, and PH-LHF is defined as post-capillary PH that mPAP ≥ 25 mmHg and pulmonary arterial wedge pressure (PAWP) > 15 mmHg at rest as assessed by RHC, pre-capillary PH is defined as mPAP ≥ 25 mmHg and PAWP ≤ 15 mmHg (2). Coronary artery disease (CAD) is defined as having at least one focus of coronary stenosis greater than 50% or having a prior physician-documented history of CAD (the data of coronary angiography performed in other hospitals are not available). The severity of CAD was evaluated using the Gensini score (12). Ischemic cardiomyopathy (ICM) is a left ventricular (LV) dysfunction with a left ventricular ejection fraction (LVEF) ≤ 40% caused by CAD (13). HFpEF is defined as LVEF ≥ 50% and HFrEF as LVEF < 50%. The estimated glomerular filtration rate (eGFR) was calculated by the Cockcroft-Gault equation (14).

All patients enrolled had two-dimensional echocardiography and RHC data. Medical histories, demographics, baseline clinical and radiograph data, laboratory results, and treatments were reviewed from our database records of the registry study.

### Endpoint and follow-up

The endpoint of this study was all-cause mortality. Follow-ups were performed using telephone calls, messages, and outpatient visits every 6 months ± 2 weeks. Patients were followed from when they were diagnosed with PH-LHF until the endpoint (death) or until this study’s cutoff date (October 2021). Patients who could not be followed up were censored at the last known follow-up data.

### Missing and extreme data

The remaining variables were interpolated using multiple imputations before entering the multivariable model for analysis. Missing data was defined as the absence of both values concurrently for variables with the same clinical significance, such as BNP and NT-proBNP. Biomarker levels below the detection limit were set to half that level, while those above the detection limit were set to the upper limit level.

### Statistical analysis

Statistical analysis was conducted using R software (version 4.0.2) and SPSS (version 24.0). Continuous variables were expressed as mean ± standard deviation for normally distributed data. In the case of skewed distributions, median with interquartile range (IQR, 25th–75th percentiles) and their differences between groups were compared with the unpaired two-tailed *t*-test or Mann–Whitney *U* test. Categorical variables were presented as counts and percentages (%). The differences between groups were compared using either Pearson’s Chi-square test (all expected values no less than 5) or Fisher’s exact test (any expected values less than 5). Logistic regression analysis was used to assess factors associated with PH-LHF. The variables identified by univariable regression models (*p* < 0.10) were then included in the multivariable logistic regression model to determine whether they could independently affect PH-LHF. The continuous variables were transformed into categorical variables determined by a median or mean in logistic regression analysis. We used the Kaplan-Meier method to estimate the cumulative incidence of the endpoint by censoring data for patients lost to follow-up. The survival analysis was described using the Kaplan-Meier survival analysis method with the log-rank test. Multivariate Cox proportional hazard regression with the forward LR (forward stepwise regression based on maximum likelihood estimation) analysis method was used to evaluate the effect of variables on survival time, yielding data as hazard ratio (HR) with a 95% CI. Variables were included in the multivariate Cox model based on clinical expertise, previous literature, and univariate analyses. The proportionality of hazards was assessed for each variable. We examined the assumption of the proportional hazards by testing the statistical significance of interactions between follow-up time and variables. Statistical significance was set at a two-sided *p*-value < 0.05.

## Results

### Baseline characteristics of left heart failure patients

A total of 480 patients with LHF were enrolled, with 106 (22.1%) patients presenting HFrEF and 374 (77.9%) presenting HFpEF. These LHF patients were predominately male (*n* = 357, 74.4%) and CAD (*n* = 378, 78.8%). Of those CAD patients, 283 (74.9%) were confirmed by angiogram, and 95 (25.1%) by a physician-documented history of CAD. The PH-LHF patient group had a higher BMI, Uric acid, NT-proBNP and a higher percentage of HFrEF, and functional class (FC) III/IV than the non-PH-LHF group (Table 1). The left atrial anteroposterior diameter (LAAPD), left ventricular end-diastolic diameter (LVEDD), right anteroposterior ventricular diameter (RVAPD), mPAP, and left ventricular end-diastolic pressure (LVEDP) were all higher in PH-LHF patients. Other baselines, demographic, clinical, and hemodynamic characteristics of patients with PH-LHF and non-PH-LHF are reported in Table 1. Missing values for the covariate variables ranged from 0.2% for LAAPD to 11.5% for Natriuretic peptides (Table 1).

**TABLE 1:** Baseline demographic, clinical and hemodynamic characteristics of all patients enrolled, patients with PH-LHF, and non-PH-LHF.

	Overall	PH-LHF	non-PH-LHF	

	(*n* = 480)	(*n* = 215)	(*n* = 265)	*p* value
Age (years)	62.9 ± 12.0	63.8 ± 12.4	62.1 ± 11.6	0.124
Female, n (%)	123 (25.6%)	63 (29.3%)	60 (22.6%)	0.115
BMI (kg/m^2^)	22.7 ± 2.6	23.2 ± 3.0	22.3 ± 2.3	0.001
**NYHA FC, n (%)**				
II	340 (70.8%)	126 (58.6%)	214 (80.8%)	<0.001
III/IV	140 (29.2%)	89 (41.4%)	51 (19.2%)	<0.001
**Types of HF**				
HFrEF, n (%)	106 (22.1%)	67 (31.2%)	39 (14.7%)	<0.001
HFpEF, n (%)	374 (77.9%)	148 (68.8%)	226 (85.3%)	<0.001
Heart rate (bpm)	75.8 ± 14.0	76.8 ± 15.2	75.0 ± 13.0	0.143
Respiratory rate (bpm)	19.2 ± 1.8	19.4 ± 1.9	19.1 ± 1.6	0.134
SBP (mmHg)	134.5 ± 22.1	132.7 ± 22.8	135.9 ± 21.4	0.114
DBP (mmHg)	76.7 ± 12.5	76.3 ± 12.1	77.1 ± 12.9	0.489
CAD, n (%)	378 (78.8%)	156 (72.6%)	222 (83.8%)	0.003
Hypertension, n (%)	214 (44.6%)	100 (46.5%)	114 (43.0%)	0.461
Hyperlipidemia, n (%) *	125 (26.0%)	60 (27.9%)	65 (24.5%)	0.405
Diabetes, n (%)	115 (24.0%)	59 (27.4%)	56 (21.1%)	0.132
Ischemic stroke, n (%)	31 (6.5%)	16 (7.4%)	15 (5.7%)	0.458
Atrial fibrillation, n (%)	22 (4.6%)	13 (6.0%)	9 (3.4%)	0.192
**Biochemistry**				
Hemoglobin (g/L)	133.1 ± 18.9	132.5 ± 20.4	133.6 ± 17.6	0.525
Platelet (× 10^9^/L)	219.7 ± 66.8	220.6 ± 70.3	218.9 ± 63.9	0.777
ALT (IU/L)	22.0 (15.0/36.5)	22.2 (15.5/37.0)	21.6 (14.5/34.9)	0.377
AST (IU/L)	22.0 (16.5/34.1)	23.2 (17.0/37.1)	21.0 (16.2/33.0)	0.122
TBil (umol/L)	11.7 (8.0/16.7)	12.0 (8.0/17.7)	11.4 (8.0/15.8)	0.361
Albumin (g/L)	40.0 ± 11.5	39.1 ± 5.3	40.7 ± 14.8	0.139
FBG (mmol/L)	5.2 (4.7/6.1)	5.4 (4.7/6.3)	5.1 (4.7/5.9)	0.113
eGFR (ml/min)	74.6 ± 27.9	72.6 ± 30.8	76.2 ± 25.2	0.169
BUN (mmol/L)	5.1 (4.0/6.8)	5.4 (4.3/7.0)	4.9 (3.8/6.5)	0.102
Uric acid (umol/L)	370.6 ± 118.0	383.6 ± 125.1	360.0 ± 110.1	0.030
**Natriuretic peptides**				
BNP (pg/Ml)	331.5 (150.6/626.5)	337.0 (185.7/649.5)	313.0 (120.0/611.9)	0.393
NT-proBNP (pg/Ml)	578.0 (120.0/1803.0)	1039.0 (235.0/2528.5)	293.0 (104.5/1330.8)	<0.001
Triglyceride (mmol/L)	1.4 (1.0/2.0)	1.4 (1.0/2.0)	1.3 (1.0/1.9)	0.461
Cholesterol (mmol/L)	4.7 (3.7/5.6)	4.5 (3.6/5.6)	4.7 (3.8/5.6)	0.598
LDL (mmol/L)	2.8 ± 1.2	2.8 ± 1.1	2.8 ± 1.3	0.796
HDL (mmol/L)	1.2 ± 0.4	1.1 ± 0.3	1.2 ± 0.4	0.014
**RHC**				
mRAP (mmHg)	13.1 ± 4.2	14.6 ± 4.2	11.9 ± 3.8	<0.001
RVSP (mmHg)	40.5 ± 12.7	47.8 ± 13.1	34.5 ± 8.5	<0.001
RVEDP (mmHg)	12.5 ± 5.6	14.0 ± 6.2	11.0 ± 4.4	<0.001
sPAP (mmHg)	40.5 ± 12.2	48.2 ± 12.2	34.2 ± 8.0	<0.001
dPAP (mmHg)	20.0 ± 6.8	24.0 ± 7.0	16.8 ± 4.5	<0.001
mPAP (mmHg)	26.0 (21.0/30.0)	30.0 (27.0/36.0)	22.0 (20.0/25.0)	<0.001
PAWP (mmHg)	19.0 (17.0/24.0)	19.0 (17.0/24.5)	18.0 (16.0/23.5)	0.235
LVEDP (mmHg)	16.0 (15.0/18.0)	17.0 (16.0/20.0)	15.0 (13.0/15.0)	<0.001
**Echocardiography**				
LAAPD (mm)	34.6 ± 6.0	36.6 ± 7.2	33.0 ± 4.3	<0.001
LVEDD (mm)	48.4 ± 7.7	50.8 ± 8.5	46.4 ± 6.2	<0.001
RVAPD (mm)	19.4 ± 4.7	20.8 ± 6.1	18.4 ± 2.9	<0.001
LVEF (%)	55.1 ± 10.0	53.3 ± 11.0	56.5 ± 8.9	0.001
Pericardial effusion, n (%)	15 (3.1%)	11 (5.1%)	4 (1.5%)	0.033
**Medications, n (%)**				
Aldactone	201 (41.9%)	125 (58.1%)	76 (28.7%)	<0.001
ACEI	211 (44.0%)	98 (45.6%)	113 (42.6%)	0.579
ARB	110 (22.9%)	50 (24.2%)	60 (23.0%)	0.826
Beta blocker	324 (67.5%)	149 (69.3%)	175 (66.0%)	0.493
Diuretic	187 (39.0%)	108 (50.2%)	79 (29.8%)	<0.001
CCB	90 (18.8%)	44 (20.5%)	46 (17.4%)	0.412
Statin	417 (86.9%)	185 (86.0%)	232 (87.5%)	0.684
Antiplatelet	400 (83.3%)	186 (86.5%)	214 (80.8%)	0.109
Anticoagulation	24 (5.0%)	12 (5.6%)	12 (4.5%)	0.676

Among the 480 patients, the amount of missing values for the covariates were: (1) (0.2%) for LAAPD, LVEDD, and LVEF; (2) (0.4%) for ALT and Albumin; (3) (0.6%) for AST, Uric acid and RVEDP; (4) (0.8%) for RVSP, TBil, TG, TC and HDL; and (5) (1.0%) for LDL and mRAP; 8 (1.7%) for FBG; 23 (4.8%) for RVAPD; 55 (11.5%) for Natriuretic peptides. CAD, coronary artery disease; BMI, body mass index; NYHA FC, New York Heart Association Functional Class; HFrEF, heart failure with reduced ejection fraction; HFpEF, left ventricular diastolic dysfunction heart failure with preserved ejection fraction; SBP, systolic blood pressure; DBP, diastolic blood pressure; PH-LHF, pulmonary hypertension due to left heart failure; ALT, alanine aminotransferase; AST, aspartate aminotransferase; TBil, total bilirubin; FBG, fasting blood glucose; eGFR, estimated glomerular filtration rate; BUN, blood urea nitrogen; BNP, b-type natriuretic peptide; NT-pro BNP, N-terminal pro b-type natriuretic peptide; LDL, low-density lipoprotein cholesterol; HDL, high-density lipoprotein cholesterol; RHC, right heart catheterization; mRAP; mean right atrial pressure; RVSP, right ventricular systolic pressure; RVEDP, right ventricular end diastolic pressure; sPAP, systolic pulmonary artery pressure; dPAP, diastolic pulmonary artery pressure; mPAP, mean pulmonary artery pressure; PAWP, pulmonary artery wedge pressure; LVEDP, left ventricular end-diastolic pressure; LAAPD, left atrial anteroposterior diameter; LVEDD, left ventricular end diastolic diameter; RVAPD, right ventricular anteroposterior diameter; LVEF, left ventricular ejection fraction; ACEI, angiotensin-converting enzyme inhibitors; ARB, angiotensin receptor blocker; CCB, calcium channel blocker. *Hyperlipidemia is defined as LDL ≥ 4.1 mmol/l or TC ≥ 6.2 mmol/l.

### Proportion of pulmonary hypertension due to left heart failure in coronary artery disease and without coronary artery disease groups

**FIGURE 2:**
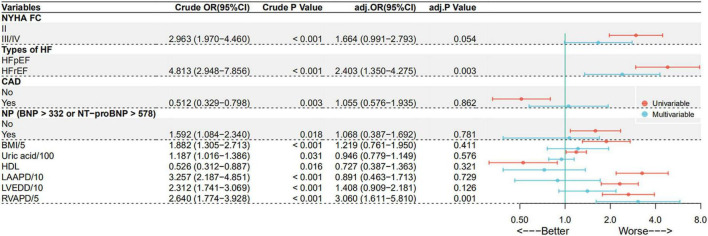
Predict factors for PH-LHF in LHF patients in univariate and multivariate logistic regression analysis.

In these LHF patients, the proportion diagnosed with PH-LHF was significantly lower in CAD group than those without CAD (41.3 vs. 57.8%, *p* = 0.003). There were no significant differences between the groups in Pre-capillary PH (15.1 vs. 14.7%, *p* = 0.925). Univariate logistic regression analysis showed that CAD is associated with a lower risk of developing PH-LHF (OR: 0.512, 95% CI: 0.329 – 0.798, *p* = 0.003) (Figure 2). However, multivariable logistic regression analysis showed that CAD is not associated with PH-LHF (Adjusted OR: 1.055, 95% CI: 0.576 – 1.935, *p* = 0.862). Only HFrEF and RVAPD were independently associated with PH-LHF (Figure 2). The results about the independent predictive factors for PH-LHF in LHF patients remained stable in the logistic regression model, including the center as a random effect (Supplementary Table 1).

### Long-term follow-up

#### Baseline characteristics of pulmonary hypertension due to left heart failure patients

Among all LHF patients enrolled, 215 patients were diagnosed with PH-LHF by RHC, with an overall proportion of 44.8%. Of those PH-LHF patients, 156 (72.6%) had CAD. The mean age and LVEF were all higher in CAD patients. Moreover, CAD group had a higher FC II and HFpEF percentage than those without CAD (Table 2). The heart rate, NT-proBNP, mPAP, LVEDP, LAAPD, and RVAPD were significantly lower in the patients with CAD (Table 2). Other baselines, demographic, clinical, and hemodynamic characteristics of PH-LHF patients with CAD and without CAD are reported in Table 2.

**TABLE 2:** Baseline demographic, clinical and hemodynamic characteristics of all patients with PH-LHF, patients with CAD and without CAD.

	Overall	Patients with CAD	Patients without CAD	

	(*n* = 215)	(*n* = 156)	(*n* = 59)	*p* value
Age (years)	63.8 ± 12.4	65.5 ± 12.2	59.4 ± 12.0	0.001
Female, n (%)	63 (29.3%)	38 (24.4%)	25 (42.4%)	0.010
BMI (kg/m^2^)	23.2 ± 3.0	22.8 ± 2.5	24.1 ± 3.7	0.013
**NYHA FC, n (%)**				
II	126 (58.6%)	98 (62.8%)	28 (47.5%)	0.041
III/IV	89 (41.4%)	58 (37.2%)	31 (52.5%)	0.041
**Types of HF**				
HFrEF, n (%)	67 (31.2%)	39 (25.0%)	28 (47.5%)	0.002
HFpEF, n (%)	148 (68.8%)	117 (75.0%)	31 (52.5%)	0.002
Heart rate (bpm)	76.8 ± 15.2	75.6 ± 14.2	80.2 ± 17.2	0.047
Respiratory rate (bpm)	19.4 ± 1.9	19.5 ± 1.8	19.2 ± 2.1	0.241
SBP (mmHg)	132.7 ± 22.8	133.8 ± 22.5	129.8 ± 23.5	0.252
DBP (mmHg)	76.3 ± 12.1	75.9 ± 11.5	77.4 ± 13.7	0.415
Hypertension, n (%)	100 (46.5%)	76 (48.7%)	24 (40.7%)	0.292
Hyperlipidemia, n (%)	60 (27.9%)	48 (30.8%)	12 (20.3%)	0.128
Diabetes, n (%)	59 (27.4%)	45 (28.8%)	14 (23.7%)	0.453
Ischemic stroke, n (%)	16 (7.5%)	11 (7.1%)	5 (8.5%)	0.773
Atrial fibrillation, n (%)	13 (6.0%)	5 (3.2%)	8 (13.6%)	0.004
**Biochemistry**				
Hemoglobin (g/L)	132.5 ± 20.4	131.4 ± 20.6	135.5 ± 19.7	0.185
Platelet (× 10^9^/L)	220.6 ± 70.3	225.9 ± 70.9	206.4 ± 67.2	0.072
ALT (IU/L)	22.2 (15.5/37.0)	22.0 (15.5/36.0)	23.2 (15.6/40.0)	0.417
AST (IU/L)	23.2 (17.0/37.1)	23.0 (15.3/38.4)	23.5 (20.1/32.3)	0.339
TBil (umol/L)	12.0 (8.0/17.7)	11.5 (8.0/16.5)	14.4 (8.6/19.4)	0.024
Albumin (g/L)	39.1 ± 5.3	39.0 ± 5.4	39.4 ± 5.0	0.604
FBG (mmol/L)	5.4 (4.7/6.3)	5.4 (4.8/6.2)	5.5 (4.6/6.3)	0.761
eGFR (ml/min)	72.6 ± 30.8	71.0 ± 28.8	77.0 ± 35.5	0.197
BUN (mmol/L)	5.4 (4.3/7.0)	5.2 (4.1/6.8)	6.2 (4.5/7.3)	0.032
Uric acid (umol/L)	383.6 ± 125.1	373.9 ± 128.6	409.1 ± 112.6	0.066
Natriuretic peptides				
BNP (pg/Ml)	337.0 (185.7/649.5)	311.0 (183.8/742.5)	346.0 (185.7/608.0)	0.692
NT-proBNP (pg/Ml)	1039.0 (235.0/2528.5)	872.0 (133.0/2014.0)	1191.0 (596.8/4280.5)	0.037
Triglyceride (mmol/L)	1.4 (1.0/2.0)	1.4 (1.0/2.2)	1.3 (0.9/1.9)	0.192
Cholesterol (mmol/L)	4.5 (3.6/5.6)	4.4 (3.5/5.6)	5.0 (4.3/5.8)	0.017
LDL (mmol/L)	2.8 ± 1.1	2.7 ± 1.1	2.9 ± 0.9	0.288
HDL (mmol/L)	1.1 ± 0.3	1.1 ± 0.3	1.2 ± 0.4	0.217
**RHC**				
mRAP (mmHg)	14.6 ± 4.2	14.8 ± 3.6	13.8 ± 5.5	0.180
RVSP (mmHg)	47.8 ± 13.1	47.0 ± 12.0	50.1 ± 15.7	0.173
RVEDP (mmHg)	14.3 ± 6.2	14.7 ± 6.2	13.3 ± 6.4	0.171
sPAP (mmHg)	48.2 ± 12.2	47.1 ± 10.9	51.0 ± 14.8	0.069
dPAP (mmHg)	24.0 ± 7.0	23.3 ± 6.1	25.9 ± 8.6	0.031
mPAP (mmHg)	30.0 (27.0/36.0)	29.0 (27.0/33.0)	33.0 (28.0/40.0)	0.015
PAWP (mmHg)	19.0 (17.0/24.5)	19.0 (16.0/23.5)	21.0 (17.3/26.8)	0.222
LVEDP (mmHg)	17.0 (16.0/20.0)	17.0 (16.0/19.0)	20.0 (16.5/24.5)	<0.001
**Echocardiography**				
LAAPD (mm)	36.6 ± 7.2	35.0 ± 4.9	40.8 ± 10.0	<0.001
LVEDD (mm)	50.8 ± 8.5	50.0 ± 7.8	52.7 ± 10.1	0.068
RVAPD (mm)	20.8 ± 6.1	19.4 ± 3.0	24.9 ± 9.8	<0.001
LVEF (%)	53.3 ± 11.0	54.6 ± 8.8	49.9 ± 14.9	0.025
Pericardial effusion, n (%)	11 (5.1%)			
**Medications, n (%)**				
Aldactone	125 (58.1%)	86 (55.1%)	39 (66.1%)	0.146
ACEI	98 (45.6%)	67 (42.9%)	31 (52.5%)	0.208
ARB	50 (23.3%)	38 (24.4%)	12 (20.3%)	0.577
Beta blocker	149 (69.3%)	110 (70.5%)	39 (66.1%)	0.531
Diuretic	108 (50.2%)	67 (42.9%)	41 (69.5%)	0.001
CCB	44 (20.5%)	31 (19.9%)	13 (22.0%)	0.726
Statin	185 (86.0%)	150 (96.2%)	35 (59.3%)	<0.001
Antiplatelet	186 (86.5%)	152 (97.4%)	34 (57.6%)	<0.001
Anticoagulation	12 (5.6%)	4 (2.6%)	8 (13.6%)	0.004

CAD, coronary artery disease; BMI, body mass index; NYHA FC, New York Heart Association Functional Class; HFrEF, heart failure with reduced ejection fraction; HFpEF, left ventricular diastolic dysfunction heart failure with preserved ejection fraction; SBP, systolic blood pressure; DBP, diastolic blood pressure; ALT, alanine aminotransferase; AST, aspartate aminotransferase; TBil, total bilirubin; FBG, fasting blood glucose; eGFR, estimated glomerular filtration rate; BUN, blood urea nitrogen; BNP, b-type natriuretic peptide; NT-pro BNP, N-terminal pro b-type natriuretic peptide; LDL, low-density lipoprotein cholesterol; HDL, high-density lipoprotein cholesterol; RHC, right heart catheterization; mRAP, mean right atrial pressure; RVSP, right ventricular systolic pressure; RVEDP, right ventricular end diastolic pressure; sPAP, systolic pulmonary artery pressure; dPAP, diastolic pulmonary artery pressure; mPAP, mean pulmonary artery pressure; PAWP, pulmonary artery wedge pressure; LVEDP, left ventricular end-diastolic pressure; LAAPD, left atrial anteroposterior diameter; LVEDD, left ventricular end diastolic diameter; RVAPD, right ventricular anteroposterior diameter; LVEF, left ventricular ejection fraction; ACEI, angiotensin-converting enzyme inhibitors; ARB, angiotensin receptor blocker; CCB, calcium channel blocker.

#### The incidence of endpoint event

**FIGURE 3:**
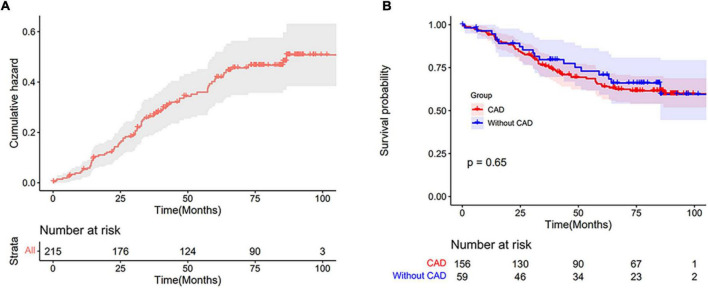
**(A)** The cumulative hazard curve of patients with PH-LHF. **(B)** Kaplan-Meier estimates of survival in PH-LHF patients with CAD and without CAD.

We conducted a long-term follow-up of patients with PH-LHF, during a median follow-up time of 84.6 months (range from 0.1 to 106.8 months), 75 patients (34.9%) died, including 35 patients suffering sudden death, 39 patients dying of advanced heart failure, and one patient dying of septic shock. In addition, five patients received percutaneous coronary intervention treatment during the follow-up. No patients underwent transplantations, coronary artery bypass grafting, implantable cardioverter defibrillator implantation or acquired any other assist devices. A total of 202 patients (94.0%) completed at least 3 years of follow-up (from enrollment to the date of death or for at least 3 years). A total of 32 patients (14.9%) were lost to follow-up. These patients could not be reached through telephone, message, hospital system, nor other possible ways for more than three times. The overall cumulative hazard curve is shown in Figure 3A. The 1-, 3-, 5-, and 8-year survival rates of all PH-LHF patients were 94.3, 76.9, 65.8, and 60.2%, respectively.

A Kaplan-Meier survival analysis showed no differences in mortality between CAD and without CAD groups (Figure 3B). HFpEF and HFrEF subgroup analysis also showed no differences in mortality between CAD and without CAD groups (Supplementary Figure 1). Sensitivity analyses were conducted to explore the impact of the missing outcome on survival. This analysis assumes that patients who lost to follow-up died or were alive. The results revealed no significant difference in mortality between CAD and without CAD (Supplementary Figure 2). The 1-, 3-, 5-, and 8-year survival rates of PH-LHF patients with CAD were 94.2, 75.9, 64.0, and 59.8%, respectively. The 1-, 3-, 5-, and 8-year survival rates of PH-LHF patients without CAD were 94.6, 79.7, 70.8, and 59.5%, respectively. Using the number of coronary arteries stenosis vessels and Gensini scores, and ICM to define CAD subgroups, we found no differences in mortality between these subgroups and without CAD group (Supplementary Figure 3).

**FIGURE 4:**
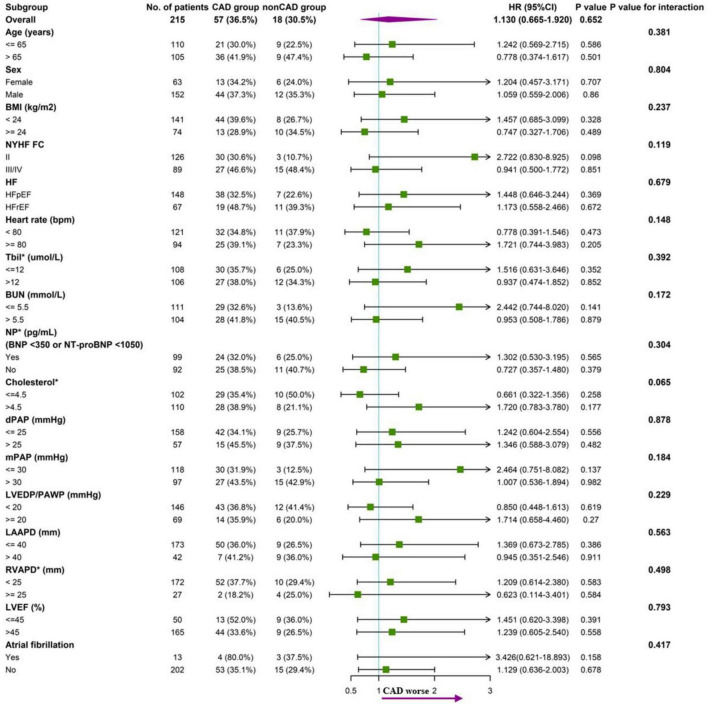
Forest plot of subgroup analysis of association between CAD and endpoint (all-cause mortality), with *p* value for interaction.

Subgroup analysis according to baseline characteristics between CAD and without CAD groups was performed. There was no trend toward an increased endpoint risk in CAD group in these subgroups. The analysis did not indicate significant interactions between the endpoint and the stratification variables (Figure 4).

#### Predictors of mortality in patients with pulmonary hypertension due to left heart failure

**FIGURE 5:**
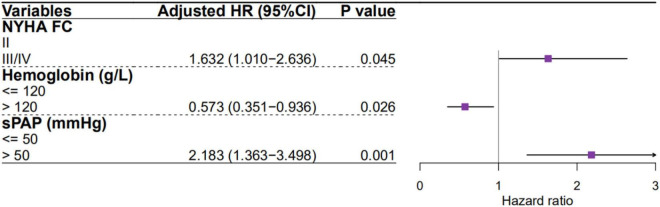
Predictors of mortality for PH-LHF patients in multivariate Cox proportional hazards regression analysis.

A univariate Cox proportional hazards regression analysis showed that age, NYHA FC, type of HF (HFpEF or HFrEF), hemoglobin, BUN, RVSP, sPAP, dPAP, and mPAP are significant predictors of mortality in PH-LHF patients. However, only NYHA FC, hemoglobin, and sPAP were significant predictors of mortality in multivariate Cox proportional hazards regression analysis (Figure 5). Predictors of mortality for PH-LHF also remained significant in the Cox proportional hazards regression model, including center as a random effect (Supplementary Table 2).

## Discussion

This study on the analysis of invasive hemodynamic data from a relatively large cohort demonstrates a high prevalence of PH in patients with LHF, and that patients with PH-LHF remain to have a poor prognosis. We found that approximately 45% of the LHF patients met the study criteria of PH-LHF when undergoing RHC (39.6% for PH-HFpEF, 63.2% for HFrEF), and a higher proportion (57.8%) in the without CAD group compared to CAD group (41.3%).

In studies based on the gold standard (RHC) as the diagnostic criteria of PH-LHF in patients, the prevalence of PH-HFpEF and PH-HFrEF were 46 and 73%, respectively (3, 15), which were relatively similar to those reported in our study (39.6 and 63.2%). Our study provides essential and reliable data to show the prevalence of PH-LHF in patients with LHF.

The pathogenesis of PH as a co-morbidity of LHF is complicated and highly heterogeneous and remains partially understood. In simple terms, the primary hemodynamic driver of PH-LHF is an impaired left ventricular (LV) systolic or diastolic function. LV dysfunction induces left atrial (LA) enlargement and reduces LA contractility and compliance. LA gradually loses ability as a buffer reservoir before the pulmonary circulation (PC), ultimately imposing increased pulsatile on the PC and leading to PH (16). At similar mean LA pressure, the LA volumes was higher and systolic function was more depressed in HFrEF when compared to HFpEF, and the global LA function (correlated with increased pulmonary vascular resistance and reduced pulmonary arterial compliance) was more impaired in HFrEF than HFpEF (17). We speculate that this may contribute to the PH-LHF development in HFrEF more than HFpEF. The current study shows that HFrEF is an independent predictive factor for PH-LHF (Adjusted OR: 2.403, 95% CI: 1.350 – 4.275, *p* = 0.003). CAD, hypertension, and dilated cardiomyopathy (DCM) are the most common causes of LHF (18). CAD is common in both HFpEF and HFrEF, (19) the etiologies of HFpEF did not include DCM, as the term DCM was defined as LVEF < 50% (20). LHF patients without CAD will have a higher chance of having DCM, making them more likely to have HFrEF. Our study showed that without CAD group had a higher percentage of HFrEF than CAD group (37.3 vs. 18.0%, *p* < 0.001). Therefore, the higher prevalence of PH-LHF in without CAD group in this study may be due to the higher percentage of HFrEF.

When PH develops in patients with LHF, it dramatically reduces their exercise capacity, aggravates symptom burden, and worsens prognosis. Even a mild increase in pulmonary artery pressure within the accepted normal range is associated with higher mortality (21–23). Previous studies reported that the 5-year of all-cause mortality of PH-LHF was between 48.5 and 52.0% (3, 24). Our study reported a lower 5-year all-cause mortality of 34.2% in patients with PH-LHF. We speculate that this is due to our study’s relatively high rate of lost follow-up (14.9%), which underestimates mortality. Nevertheless, our finding still supports the overall poor prognosis of PH-LHF. It is generally accepted that the different etiologies or co-morbidities in LHF patients have distinct mortality. Previous studies reported that HFrEF of ischemic origin had higher mortality than HFrEF due to non-ischemic causes (25, 26). A study exploring the prognosis in patients with HFpEF reported a specific phenogroup containing all patients with CAD had significantly different mortality compared with other phenogroups (27). However, our study revealed no significant differences in mortality between CAD and without CAD groups in HFrEF and HFpEF (Supplementary Figure 2). Repeating the analysis using ICM and without CAD groups gave the analysis almost identical results. In addition, the results remained stable in the subgroup analysis (Figure 4). Therefore, we speculate that the effect of CAD on the long-term outcome of patients with LHF may disappear once they develop to PH-LHF.

Heart failure with reduced ejection fraction is generally considered to confer a worse survival than HFpEF. However, several observational studies show that this difference is negligible (28, 29). Recently, a study exploring long-term outcomes in LHF patients reported that mortality was independent of HF (HFrEF or HFpEF), while NYHA FC was a significant predictor of all-cause mortality (30). Similarly, our study shows that types of HF were not an independent predictor of mortality in PH-LHF patients, while NYHA FC was (Figure 5). Agarwal et al. also reported that FC was an independently predicted mortality factor in PH-LHF (31). It indicates that we should pay more attention to the FC, rather than the type of HF, during the follow-up period of PH-LHF patients.

Anemia is a highly prevalent co-morbidity in patients with LHF, affecting about one-third of LHF patients (32). Similarly, the prevalence of anemia in LHF patients was 32.9% in our study. The presence of anemia is independently associated with poor FC and increased all-cause mortality in patients with LHF (33, 34). The present study also reported that the reduction in hemoglobin is accompanied by a significantly increased risk of mortality in PH-LHF patients (Figure 5). The mechanism of the increased mortality among LHF patients with anemia may be due to chronic tissue hypoxia (33). Therefore, we suggest that all PH-LHF patients with anemia should be investigated for the underlying etiologies and treated according to the current guidelines.

We found that the sPAP is the most potent risk factor for mortality in PH-LHF patients (Figure 5). Previous studies have also reported that the sPAP was associated with mortality in PH-LHF (9, 15). This suggests that pulmonary artery pressure should be closely monitored in patients with PH-LHF while actively treating the underlying LHF. Although selective pulmonary vasodilators are still not recommended in PH-HF (2, 35), some distinct subgroups still benefit from the selective pulmonary vasodilators (36). Future studies should focus on patients whose left heart function has improved (PAWP ≤ 15 mmHg) after treatment of the LHF, but pulmonary artery pressure remains elevated to screen out PH-LHF patients sensitive to selective pulmonary vasodilators.

## Study limitations

While interpreting these results, several issues should be considered. First, this is a retrospective analysis of a prospective cohort study with inherited limitations, including possible selection bias. Second, the rate of lost follow-up was 14.9% in our study. However, the results remained stable in the sensitivity analysis. Finally, the parameter of pulmonary vascular resistance is not available. Thus, the two forms of PH-LHF (isolated post-capillary PH and combined post-capillary and pre-capillary PH) could not be distinguished.

## Conclusion

Group 2 PH is commonly identified in patients with LHF and its prevalence is lower in patients with CAD than that without CAD. The mortality is still high in patients with PH-LHF, highlighting that the development of risk table to screen high-risk patients for closer monitoring and intensification of management may improve prognosis. FC, hemoglobin, and sPAP are independent risk predictors of mortality for PH-LHF. These findings may be useful for risk stratification in future clinical trial enrollment.

## Data availability statement

The raw data supporting the conclusions of this article will be made available by the authors, without undue reservation.

## Ethics statement

The studies involving human participants were reviewed and approved by the Institutional Review Board of Fuwai Hospital. The patients/participants provided their written informed consent to participate in this study.

## Author contributions

YL and JH: contributing to the conception and design. YL: drafting the manuscript. LP, SH, JSh, WW, FT, XZ, WS, JSu, and TY: data collection, analysis, and interpretation. RQ and HH: revising the manuscript. All authors contributed to the article and approved the submitted version.
