# Supplementary material for: The prevalence and survival of pulmonary hypertension due to left heart failure: A retrospective analysis of a multicenter prospective cohort study
Source: Front Cardiovasc Med. 2022 Aug 2;9:908215. doi: 10.3389/fcvm.2022.908215 (PMC9378855; doi:10.3389/fcvm.2022.908215)
Supplement: Supplementary file 1 [file Data_Sheet_1.pdf]

Supplementary materials

Supplementary Figure 1. (A) Kaplan-Meier estimates of survival in PH-HFpEF patients with CAD and without CAD. **(B)** Kaplan-Meier estimates of survival in PH-HFrEF patients with CAD and without CAD.

Supplementary Figure 2. Kaplan-Meier estimates of survival in PH-LHF patients with CAD and without CAD that assumes patients who lost to follow-up all died **(A)**, or all alive **(B)**.

Supplementary Figure 3. (A) Kaplan-Meier estimates of survival in patients with CAD as defined by number of coronary artery stenosis vessels (Unknown, Single-vessel, Two-vessels, Three-vessels) and without CAD. **(B)** Kaplan-Meier estimates of survival in patients with CAD as defined by Gensini scores (Unknown, 2 – 40, 41 – 80, > 80) and without CAD. **(C)** Kaplan-Meier estimates of survival in patients with CAD as defined by ischemic cardiomyopathy (ICM) (with or without ICM) and without CAD.

Supplementary Table 1. Predict factors for PH-LHF in LHF patients in univariate and multivariate logistic regression model including center as random effect.

Variables	Crude OR (95%CI)	Crude P value	Adj. OR (95%CI)	Adj. P value
BMI/5	1.897 (1.282-2.808)	0.013	1.297 (0.840-2.010)	0.240
NYHA FC				
II				
III/IV	2.574 (1.688-3.924)	< 0.001	1.618 (0.990-2.638)	0.054
Types of HF				
HFpEF				
HFrEF	3.938 (2.379-6.519)	< 0.001	2.317 (1.340-4.033)	0.003
CAD				
No				
Yes	0.732 (0.449-1.191)	0.209	1.083 (0.622-1.885)	0.779
HDL	0.545 (0.318-0.935)	0.028	0.656 (0.364-1.183)	0.162
LAAPD/10	2.749 (1.746-4.328)	< 0.001	0.984 (0.551-1.759)	0.958
LVEDD/10	2.069 (1.533-2.792)	< 0.001	1.417 (0.953-2.106)	0.085
RVAPD/5	2.640 (1.774-3.928)	< 0.001	2.137 (1.316-3.480)	0.002

PH-LHF, pulmonary hypertension due to left heart failure; LHF, left heart failure; Adj. OR, adjusted odds ratio; BMI, body mass index; NYHA FC, New York Heart Association Functional Class; HFrEF, heart failure with reduced ejection fraction; HFpEF, left ventricular diastolic dysfunction heart failure with preserved ejection fraction; CAD, coronary artery disease; HDL, high-density lipoprotein cholesterol; LAAPD, left atrial anteroposterior diameter; LVEDD, left ventricular end diastolic diameter; RVAPD, right ventricular anteroposterior diameter.

Supplementary Table 2. Predictors of mortality for PH-LHF patients in Cox proportional hazards regression model including center as random effect.

Variables	Crude HR (95%CI)	Crude P value	Adj. HR (95%CI)	Adj. P value
Age				
≤65				
>65	1.746 (1.099-2.773)	0.018		
NYHA FC				
II				
III/IV	2.116 (1.340-3.340)	0.001	1.627 (1.007-2.627)	0.047
HF				
HFpEF				
HFrEF	1.747 (1.100-2.775)	0.018		
CAD				
No				
Yes	1.166 (0.648-2.100)	0.608		
Hemoglobin				
≤120				
>120	0.490 (0.303-0.793)	0.004	0.574 (0.351-0.938)	0.027
BUN				
≤5.5				
>5.5	1.654 (1.046-2.617)	0.031		
sPAP				
≤50				
>50	2.543 (1.613-4.010)	<0.001	2.177 (1.359-3.488)	0.001
dPAP				
≤25				
>25	1.635 (1.006-2.658)	0.047		
mPAP				
≤30				
>30	1.789 (1.134-2.825)	0.012		
Af				
No				
Yes	1.689 (0.775-3.680)	0.188		

PH-LHF, pulmonary hypertension due to left heart failure; Adj. HR, adjusted hazard ratio; NYHA FC, New York Heart Association Functional Class; HFrEF, heart failure with reduced ejection fraction; HFpEF, left ventricular diastolic dysfunction heart failure with preserved ejection fraction; CAD, coronary artery disease; BUN, blood urea nitrogen; sPAP, systolic pulmonary artery pressure; dPAP, diastolic pulmonary artery pressure; mPAP, mean pulmonary artery pressure; Af, atrial fibrillation.

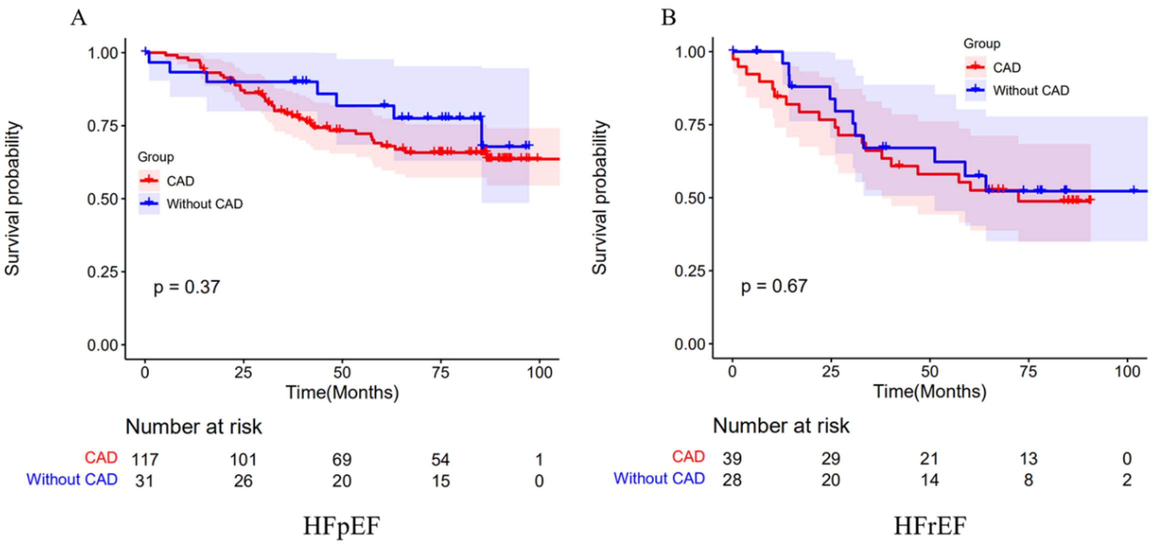

Supplementary Figure 1. (A) Kaplan-Meier estimates of survival in PH-HFpEF patients with CAD and without CAD. **(B)** Kaplan-Meier estimates of survival in PH-HFrEF patients with CAD and without CAD.

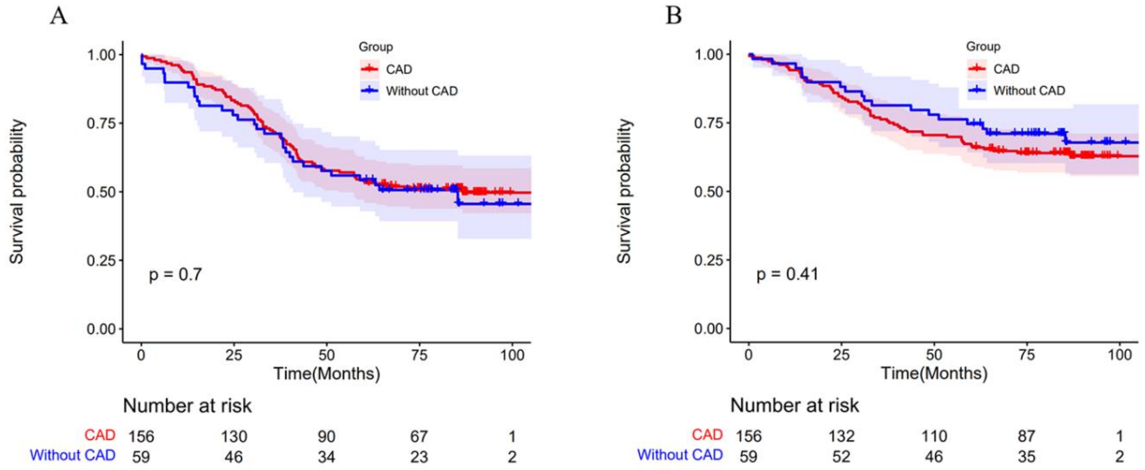

Supplementary Figure 2. Kaplan-Meier estimates of survival in PH-LHF patients with CAD and without CAD that assumes patients who lost to follow-up all died **(A)**, or all alive **(B)**.

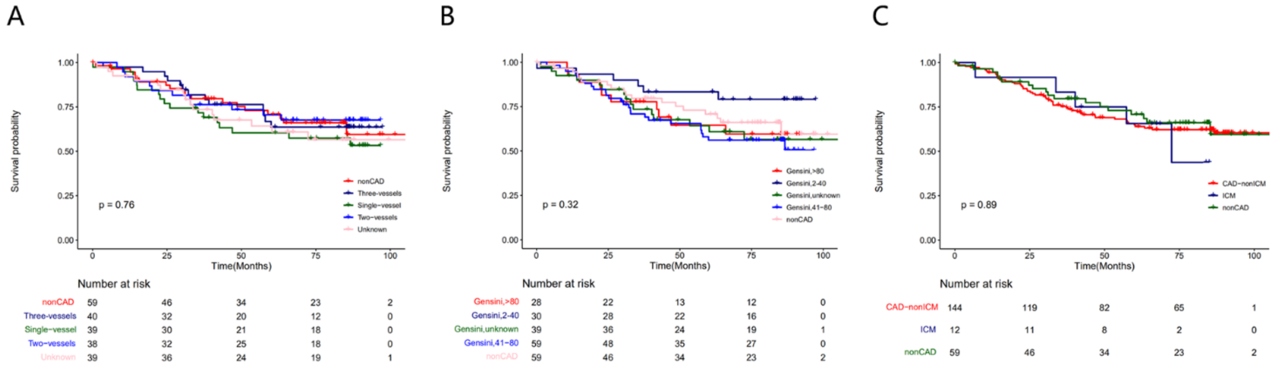

Supplementary Figure 3. (A) Kaplan-Meier estimates of survival in patients with CAD as defined by number of coronary artery stenosis vessels (Unknown, Single-vessel, Two-vessels, Three-vessels) and without CAD. **(B)** Kaplan-Meier estimates of survival in patients with CAD as defined by Gensini scores (Unknown, 2 – 40, 41 – 80, > 80) and without CAD. **(C)** Kaplan-Meier estimates of survival in patients with CAD as defined by ischemic cardiomyopathy (ICM) (with or without ICM) and without CAD.
